# Functionally Active Microheterogeneous Systems for Elastomer Fire- and Heat-Protective Materials

**DOI:** 10.3390/molecules28135267

**Published:** 2023-07-07

**Authors:** Victor F. Kablov, Oksana M. Novopoltseva, Daria A. Kryukova, Natalia A. Keibal, Vladimir Burmistrov, Vladimir G. Kochetkov

**Affiliations:** 1 Department of Chemical Technology of Polymers and Industrial Ecology, Volzhsky Polytechnic Institute (Branch) of Volgograd State Technical University, 42a Engelsa st., Volzhsky 404121, Russia; 2 Department of Organic Chemistry, Volgograd State Technical University, 28 Lenina Avenue, Volgograd 400005, Russia; crus_himself@mail.ru

**Keywords:** microspheres, microfibers, elastomers, fire- and heat-protective material, organoelement modifiers

## Abstract

Elastomeric materials are utilized for the short-term protection of products and structures operating under extreme conditions in the aerospace, marine, and oil and gas industries. This research aims to study the influence of functionally active structures on the physical, mechanical, thermophysical, and fire- and heat-protective characteristics of elastomer compositions. The physical and mechanical properties of elastomer samples were determined using Shimazu AG-Xplus, while morphological research into microheterogeneous systems and coke structures was carried out on a scanning electronic microscope, Versa 3D. Differential thermal and thermogravimetric analyses of the samples were conducted on derivatograph Q-1500D. The presence of aluminosilicate microspheres, carbon microfibers, and a phosphor–nitrogen–organic modifier as part of the aforementioned structures contributes to the appearance of a synergetic effect, which results in an increase in the heat-protective properties of a material due to the enhancement in coke strength and intensification of material carbonization processes. The results indicate an 8–17% increase in the heating time of the unheated surface of a sample and a decrease in its linear burning speed by 6–17% compared to known analogues. In conclusion, microspheres compensate for the negative impact of microfibers on the density and thermal conductivity of a composition.

## 1. Introduction

The development of elastomer materials based on ethylene–propylene–diene rubber that can withstand high-temperature heat flows in a short amount of time is crucial for protecting structures in industries such as aerospace, rocket, and oil and gas [1,2]. These materials can be used as coatings for fire and heat protection in combustion chambers, nozzles of solid-propellant rocket engines, gas generator covers, and other applications.

Advancements in creating fire-resistant polymer materials have been explored by Walter, Bhuvaneswari, and Ahmed [3,4,5]. The effectiveness of elastomeric fire- and heat-protective materials (FHPMs) largely depends on a complex set of endothermic physical and chemical transformations of components and their thermal destruction, as well as changes in the material’s chemical structure, such as intumescence, pore formation, and carbonization [6,7].

Intensive thermal impact on FHPMs initiates structurization and carbonization processes that lead to the formation of a protective layer with low thermal conductivity [6,7]. In most heat-protective compositions, non-organic fillers act as elements on which primary carbon appears and then coke deposits as a result of thermal decomposition. Introducing phosphorus-, boron-, or nitrogen-containing components can intensify carbonization processes. Microspheres can also be introduced to create a uniform, fine porous structure of the coke layer [7,8,9].

One challenge faced in FHPMs is the thinning of the material due to high-speed gas flow, which leads to a decrease in efficiency under operational conditions. This challenge can be solved by using microfiber fillers such as kaolin, basalt, carbon, and other fibers. However, introducing such fillers is associated with technical difficulties such as agglomeration and an increase in density and thermal conductivity. Various coupling agents can be used to improve the distribution of microfibers and enhance the interaction at the polymer–microfiber interface. A phosphorus-nitrogen-containing modifier (DDF) has been shown to have a positive impact on physical and mechanical, fire- and heat-protective, and thermophysical properties of a composition [10].

Research has shown that phosphorus-containing additives can inhibit flames by increasing the recombination speed of H^+^ and OH^−^ and reactions with phosphorus oxides and oxyacids [11,12,13]. In the condensed phase, residues of phosphoric acid are formed, which act as a dehydrating agent, enabling the formation of carbonized structures. Phosphorus compounds and their degradation products can also act as cross-linking agents, causing dehydration, cyclization, ligation, aromatization, and graphitization [14,15,16].

Microcapsulated phosphorus can also be used as a modifying agent, increasing fire resistance in epoxipolymers without affecting physical and mechanical or dielectric parameters [16].

Under operating conditions, fire- and heat-resistant materials are exposed not only to elevated temperatures and pressure but also to high-speed gas flow. This leads to the removal of the upper layer of the material, thinning the material itself and decreasing its efficiency. This issue can be resolved by incorporating microfiber fillers that form a “network” to increase the erosion resistance of the material. Examples of such fillers include kaolin, basalt, carbon, and other fibers. The introduction of these fillers into polymers involves various interactions at the polymer–filler interface that affect the mechanical, physicochemical properties, and thermooxidative stability of the composite material [17,18,19,20,21].

However, the introduction of such fillers comes with a number of technological difficulties. Fibers, especially asbestos, tend to agglomerate, which reduces the homogeneity of the material. Additionally, there is an increase in the density and thermal conductivity of the material, which is unacceptable in some cases.

To improve the distribution of microfibers and enhance the interaction at the polymer–microfiber interface, various finishes can be used. One criterion for selecting a sizing system is its positive effect on not only the physical and mechanical properties but also on the fire and heat protection and thermophysical properties of the composition. As our study has shown, the DDP modifier (dimethylcarbamyl(diaminomethyl)phosphoramide) synthesized by us can be such a system.

To compensate for the negative effect of microfibers on density and thermal conductivity, their combination with microspheres is possible [22,23,24,25,26]. By selecting sizing systems and varying the ratio of microspheres and microfibers, it is possible to obtain in situ organizing structures that combine the advantages of both components. The efficiency of fire- and heat-resistant elastomeric materials is primarily determined by a complex set of endothermic physical and chemical transformations of the components, as well as their thermal degradation. Additionally, the material’s chemical structure undergoes changes such as swelling, pore formation, and coking. Under high-temperature conditions, these processes are initiated, leading to the formation of a protective layer with low thermal conductivity through coke formation and structuring [25,26,27,28,29].

## 2. Results

**Figure 1:**
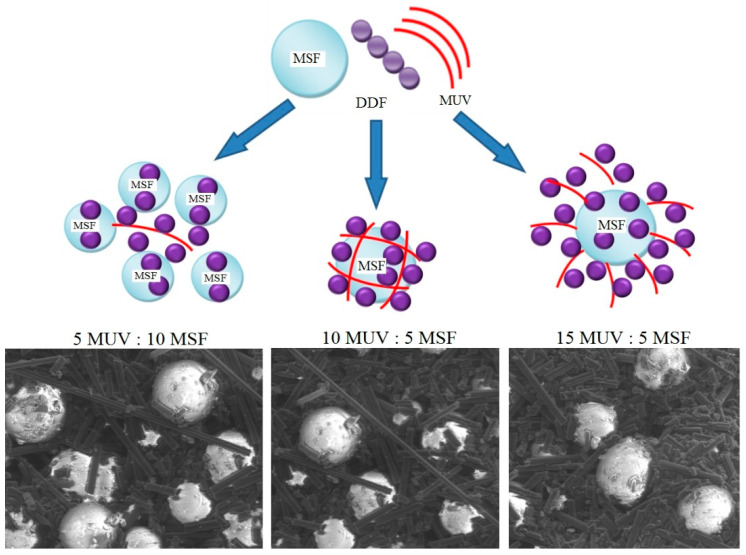
Diagram of the assembly of functionally active structures and SEM microphotos thereof (magnification× 10,000).

Depending on the proportions of microspheres and microfibers used, we anticipated the formation of various functionally active structures. Table 1 outlines the proportions of microspheres, microfibers, and DDF that were studied (Supplementary Materials). Scanning electronic microscopy revealed the formation of three distinct types of structures (as depicted in Figure 1), indicating the modification of the surface of the microdispersed components through the appearance of phosphorus atom peaks in the elementograms.

**Figure 2:**
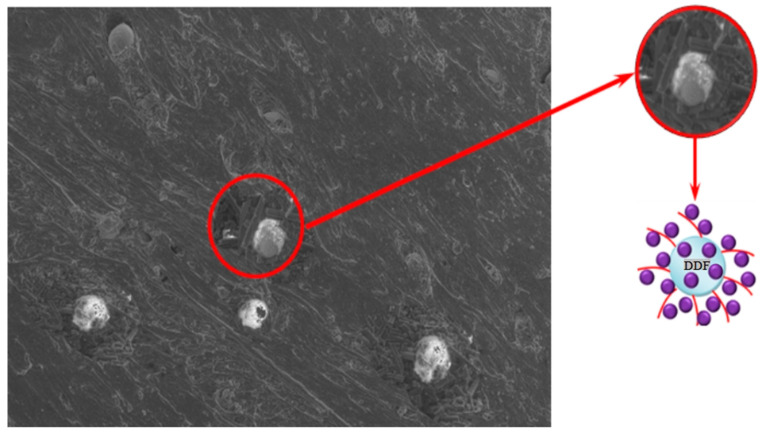
SEM microphotograph of vulcanized stock 10MUV: 5MSF (magnification× 10,000).

As illustrated in the microphotograph (Figure 2), the introduced functionally active structures (FASs) retain their integrity. The existing intermediate variants in the form of treated microspheres or microfibers also positively influence the fire and heat protection characteristics of the material: the heating time of the unheated surface of a sample up to 100 °C increases to 5.5–7.5%; the linear burning speed decreases to 4.3–6.7%.

Table 2 presents the properties of vulcanized samples of the elastomeric compositions under study.

## 3. Discussion

The introduction of functionally active components, which form relatively large microstructures, results in a certain decrease in the strength characteristics. However, their values still remain higher than the standard ones (Table 2). More importantly, the density of the compositions decreases with an increase in the content of microspheres and approaches the control variant. A decrease in this parameter is crucial for creating protective materials for aircraft and rocketry.

Previous studies [10,30] have demonstrated that the optimal balance between fire and heat protection and the physical and mechanical properties of the material can be achieved by incorporating 5–15 mass parts of microfibers and 3–7 mass parts of microspheres.

**Figure 3:**
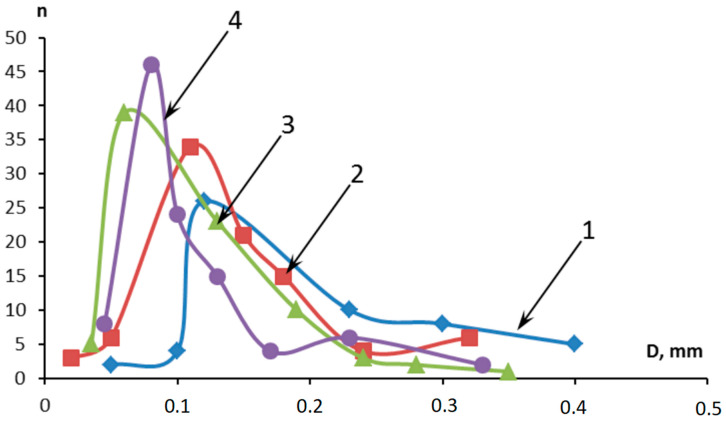
Pore distribution in the pre-pyrolysis layer: 1—control sample; 2—5MUV:10MSF; 3—10MUV:5MSF; 4—15MUV:5MSF.

Samples containing FASs with a ratio of microspheres/microfibers = 5:10 exhibit the most efficient fire and heat protection characteristics. When heat flow passes through the thickness of a fire- and heat-protective material, a number of adaptive processes occur: the destruction of the polymer matrix occurs in the upper layers of the material, and the pre-introduced functionally active components enable the formation of a denser fine porous coke layer (Figure 3), which is reinforced with microfibers. In the deeper levels of the material, carbonization processes are initiated owing to the presence of a phosphorus–boron–organic modifier on the surface of the microspheres. These processes enable a decrease in the heating-up speed.

The efficiency of the additives researched is confirmed with the DTA and TG analysis methods (Supplementary Materials). The value of the energy consumed for the structurization processes, carbonization of the material, degradation, and chemical transformation of the modifier under the impact of a heat flow can be evaluated by the square of the endothermic peak on the DTA curve. When FASs are introduced, an increase in the carbon residue occurs at 4–30% and a growth in the square of the endothermic peak takes place at 24%.

**Figure 4:**
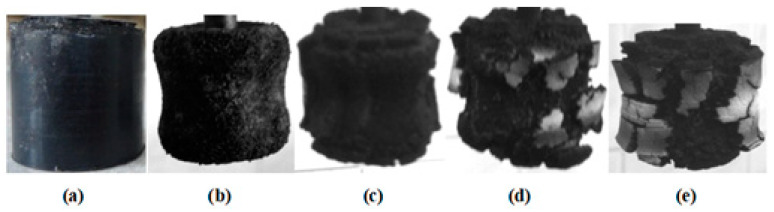
The appearance of the sample surface under the impact of high-speed heat flow: (**a**) the control sample before testing; (**b**) the control sample after testing; (**c**) 5MUV:10MSF; (**d**) 10MUV:5MSF; (**e**) 15MUV:5MSF.

The studied samples are shown in Figure 4 after being tested for erosion strength under conditions of high-speed heat flow. The control sample (Figure 4b) is characterized by greater weight loss and little start time of combustion. The presence of FASs intensifies the carbonization processes and the microfibers enable the creation of strong coke with low thermal conductivity (Figure 4e).

**Figure 5:**
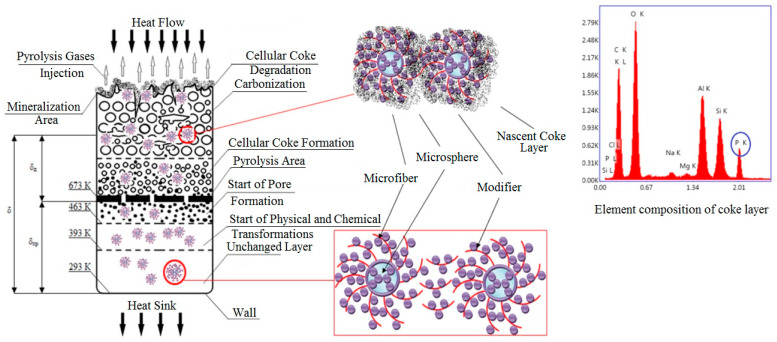
The presumed mechanism of fire- and heat-protective action of materials with the content of functionally active structures.

The experimental data obtained have allowed us to propose a mechanism for the fire- and heat-protective action of materials containing functionally active structures (as depicted in Figure 5). The introduced functionally active components are transformed into a reinforced coke layer with increased strength against erosion carry-over and decreased thermal conductivity under the impact of high-temperature heat flow. In this case, the microspheres act as carbonization centers, while a layer of DDF on their surface initiates this process and retains the microfibers necessary to enhance the coke strength under the conditions of material erosion carry-over.

**Figure 6:**
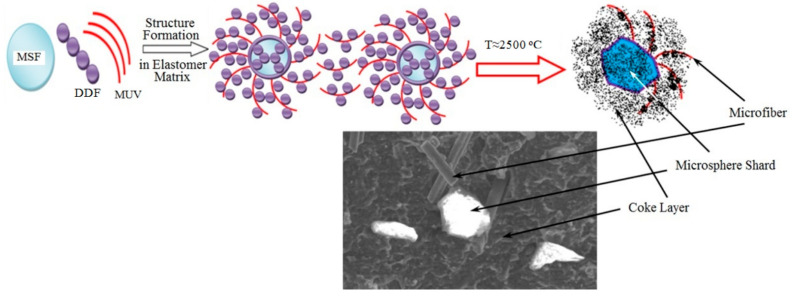
SEM microphotograph of the coke layer being formed after a high-temperature impact.

When subjected to high-temperature impact in the carbonization area, fusion and destruction of the microspheres occur. However, microfibers remain even on the surface of the shards (Figure 6), continuing to perform their function of reinforcing the coke.

## 4. Materials and Methods

### Materials

The materials under investigation were rubbers based on triple ethylene–propylene–diene rubber (EPDM-40), manufactured by the Nizhnekamsk Synthetic Rubber Factory. This particular rubber was selected for its relatively low density and unsaturation degree, as well as its high heat resistance [31].

The compositions being studied are listed in Table 3, with previous works [7,10] determining the optimal weight content of microspheres, microfibers, and an organic element modifier at 5, 10, and 1 wt pts., respectively, per 100 wt pts of rubber.

Elastomer compositions without functionally active additives were used as control samples. The properties of compositions containing single components of functionally active structures were previously studied by our team [30,32].

The rubber mixes were prepared in two stages, using a high-speed laboratory micromixer of the “Brabender” type (Polimermash, Saint Petersburg, Russia) for the first stage. A calculated amount of the functionally active modifier was added to the compounded masterbatch at a temperature of 105–110 °C and homogenized. In the second stage, sulfur, vulcanization accelerators, and activators were added on laboratory rollers (Polimermash, Saint Petersburg, Russia) at a temperature of 45–50 °C after a 24 h rest. Vulcanization of the samples was then performed in a PHG-2 212/4 vulcanizing press (Carver, Aldridge, Hungary) under the optimum mode determined using a flow meter MDR 3000 Professional (MonTech, Columbia City, IN, USA) (165 °C, 40 min.).

The physical and mechanical properties of standard elastomer samples were determined using a tearing machine Shimazu AG-Xplus 1.0 kN (Shimadzu, Kyoto, Japan) in accordance with ISO 37-2017 [33]. To evaluate the fire and heat resistance of the samples, the temperature dependence on the unheated surface of the sample from the time of open plasma torch fire impact, the loss of sample weight, and the linear burning speed were determined according to a developed procedure. The sample used was a disc with a diameter of 100 mm and thickness of 10 mm. High-temperature heating created a temperature around 2000 °C on the surface of the sample.

To evaluate the erosion strength of the material under high-temperature conditions, a sample was placed on a rotating shaft and tangentially heated by a plasma torch. The start time of combustion and coke detachment were recorded during the test, followed by the determination of the sample diameter after the test. The disrupted layer was removed, and the thickness of the non-disrupted layer was determined.

The strength characteristics of the coke were determined by considering the tear-off forces realized on the interface between the coke layer and non-degraded material during the tear-off of the coke layer under the impact of centrifugal forces when a cylindrical sample was rotated at a constant speed during high-temperature heating in a plasma torch flame. The tear propagation strength of the carbonized layer was determined using Formula (1):

(1) $$\sigma =\frac{2\cdot {\left(\pi \cdot \omega \right)}^{2}\cdot \left(R_{0}^{2}-R^{2}\right)\cdot \rho_{Κ}}{{\left(R_{0}^{}-R^{}\right)}^{2}}$$

where *ω*—angular velocity, rps; *R*_0_—initial radius, mm; *R*—radius before the boundary of the pyrolysis layer, along which the tear-off occurs, and mm; *ρ_k_*—coke density, kg·m^−3^.

**Figure 7:**
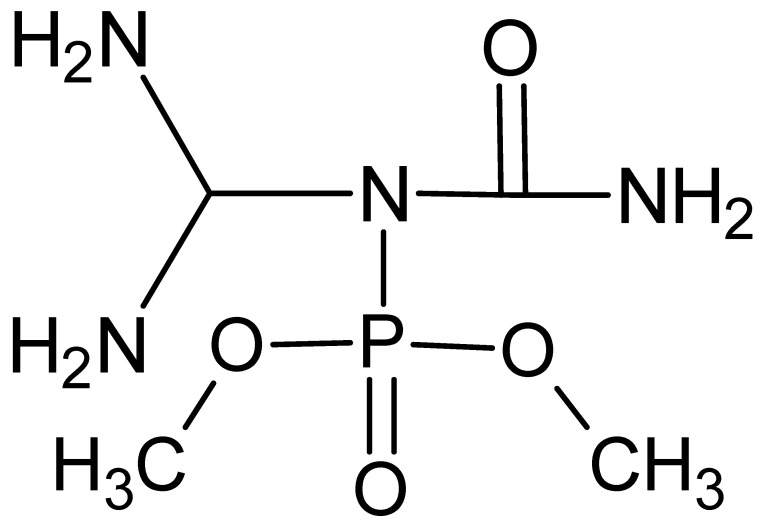
Structural formula of dimethylcarbamyl(diaminomethyl)phosphoramide [34].

The preassembly of a functionally active structure was carried out by treating the surface of the microspheres and microfibers with a 5% aqueous solution of DDF (Figure 7), followed by drying until constant weight.

Morphological research of the FAS, vulcanized stocks, and coke structures was carried out on the scanning electronic microscope Versa 3D (FEI Company, Hillsboro, OR, USA). Differential thermal and thermogravimetric analyses of the samples were carried out on a derivatograph Q-1500D (MOM Szerviz Kft, Budapest, Hungary).

## 5. Conclusions

The results indicate that incorporating pre-assembled functionally active systems into the composition of elastomeric fire- and heat-protective materials enhances their interaction with the elastomeric matrix, resulting in better distribution. This targeted delivery of the modifier into the interphase layer enhances the coke formation processes at the interface. The combined introduction of microspheres and microfibers during initiated coke formation creates structures where microfibers are grouped around the microspheres, reinforcing the coke layer and increasing its erosion resistance. Morphological analysis of the FAS, vulcanized stocks, and coke structures was conducted using the scanning electronic microscope Versa 3D. Differential thermal and thermogravimetric analyses of the samples were carried out using a derivatograph Q-1500D.

## Figures and Tables

**Table 1:** Proportions studied of microspheres, microfibers, and DDF.

Ingredient	Sample Number		
	5MUV:10MSF	10MUV:5MSF	15MUV:5MSF
	Content, wt. pts. per 100 wt. pts. Rubber		
Aluminosilicate microspheres	10	5	5
Carbon microfibers	5	10	15
DDF	1	1	1

**Table 2:** Vulcanized Rubber Properties.

Parameter	Sample Number				
	Ref.	Control Sample	5MUV:10MSF	10MUV:5MSF	15MUV:5MSF
Tensile strength *f_t_*, MPa	Not less than 6.0	16.5	8.5	11.8	12.4
Breaking elongation ε_rel_, %	Not less than 300	450	400	350	380
Permanent elongation θ_perm_, %	Not more than 30	20	25	18	18
Density ρ, kg m^−3^	Not more than 1100	1080	1065	1082	1105
Heating time of unheated surface of a sample up to 100 °C, s	–	62	70	87	83
Coke number CCV, %	–	2.4	14.8	15.9	16.7
Linear burning speed *V_l.b._*, mm min^−1^	–	32.1	30.4	26.7	25.4
Coke layer tear propagation strength σ, mPa	–	37.3	40.1	41.4	41.6

**Table 3:** Rubber Formulas.

Ingredient	Sample Number			
	Control Sample	5MUV:10MSF	10MUV:5MSF	15MUV:5MSF
	Content, wt. pts. per 100 wt. pts. Rubber			
EPDM-40	100	100	100	100
BS-120	30	30	30	30
Zinc oxide	5	5	5	5
Stearine	1	1	1	1
Captax	2	2	2	2
Sulphur	2	2	2	2
FAS	0	16	16	16
Total	140	156	156	156

## Data Availability

The data presented in this study are available in Supplementary Materials.

## Supplementary Materials

The following supporting can be downloaded at: https://www.mdpi.com/article/10.3390/molecules28135267/s1. Raw data of DTA and TG analyzes of the studied samples.

## Sample Availability

Not available.
